# A circular model for song motor control in *Serinus canaria*

**DOI:** 10.3389/fncom.2015.00041

**Published:** 2015-04-07

**Authors:** Rodrigo G. Alonso, Marcos A. Trevisan, Ana Amador, Franz Goller, Gabriel B. Mindlin

**Affiliations:** ^1^Physics Department, Facultad de Ciencias Exactas y Naturales, University of Buenos Aires and IFIBA ConicetBuenos Aires, Argentina; ^2^Department of Biology, University of UtahSalt Lake City, UT, USA

**Keywords:** non-linear dynamics, rate models, birdsong, song system, motor control

## Abstract

Song production in songbirds is controlled by a network of nuclei distributed across several brain regions, which drives respiratory and vocal motor systems to generate sound. We built a model for birdsong production, whose variables are the average activities of different neural populations within these nuclei of the song system. We focus on the predictions of respiratory patterns of song, because these can be easily measured and therefore provide a validation for the model. We test the hypothesis that it is possible to construct a model in which (1) the activity of an expiratory related (ER) neural population fits the observed pressure patterns used by canaries during singing, and (2) a higher forebrain neural population, HVC, is sparsely active, simultaneously with significant motor instances of the pressure patterns. We show that in order to achieve these two requirements, the ER neural population needs to receive two inputs: a direct one, and its copy after being processed by other areas of the song system. The model is capable of reproducing the measured respiratory patterns and makes specific predictions on the timing of HVC activity during their production. These results suggest that vocal production is controlled by a circular network rather than by a simple top-down architecture.

## Introduction

**Figure 1:**
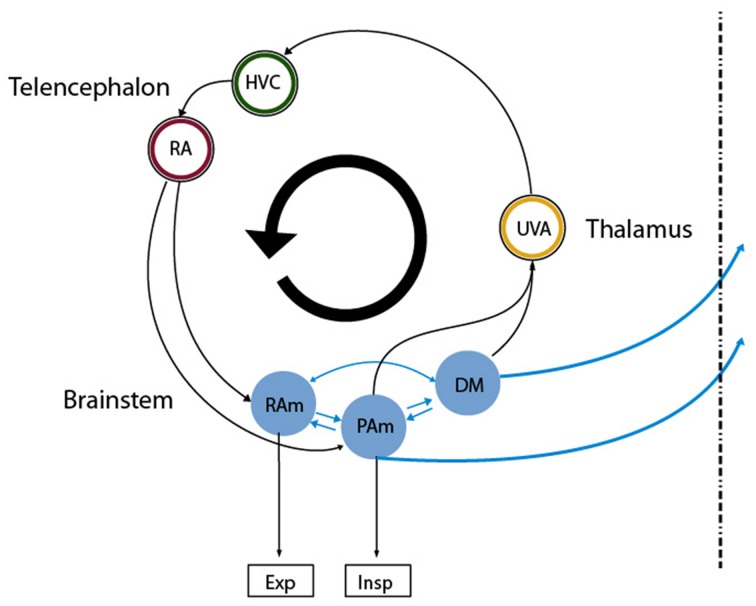
**Diagram of the avian song system**. Representation of the song system as a circular, highly interconnected set of brain areas. We used the same color code as in **Figure 3** for the neural nuclei of our model so that they can be mapped directly onto nuclei in this architecture. Nuclei in the brainstem are more difficult to map into our model, as all the reported recordings in those nuclei were performed in sleeping or anesthetized conditions. Excitatory and inhibitory populations in our model could be mapped into the analogous populations in RAm, associated with expiratory related activity. Whether the initiating area in our model is a subpopulation of either PAm or DM is not clear. In any case, the model predicts that at least one of those is likely to present neurons branching to thalamus and medulla. Both connect indirectly to cortical-like areas via the thalamic nucleus Uva, which in **Figures 3B–E** corresponds to the circle enclosing the blue ring. This starts the indirect path from the initiating area back to the one with expiratory related activity.

One of the fundamental problems in motor control is how the instructions for controlling the peripheral effectors are encoded in the central nervous system (Churchland et al., 2012). Birdsong production is a complex behavior that presents strong advantages for exploring this issue. First, the peripheral structures generating the behavior are sufficiently understood (Suthers et al., 1999), thus allowing assessment of the output of the central nervous system. This output is the set of time dependent parameters that control the respiratory rhythm and the vocal organ, the syrinx. Second, the neural brain circuitry for generating these instructions is dedicated to vocal behavior and is well characterized. The song production circuit (Figure 1) involves telencephalic areas HVC (used as proper name) and the robust arcopallial nucleus (RA), which projects to the respiratory nuclei n. retroambigualis (RAm) and n. parambigualis (PAm) and the syringeal motor nucleus (nXIIts). These hindbrain structures are connected to n. uvaeformis (Uva) and the dorsomedial nucleus (DM), which in turn provide a direct or indirect connection to HVC (Ashmore et al., 2005). In this system we can therefore systematically study how different parts of the brain participate in birdsong production.

One proposed model states that forebrain areas take control of respiratory pattern generators. In particular this model postulates that nearly all the temporal features of song are encoded in the sparse bursting of projecting neurons in the telencephalic nucleus HVC (Fee et al., 2004; Long and Fee, 2008; Andalman et al., 2011). Observation of the distinct activity patterns of different projection neurons in HVC (Hahnloser et al., 2002) show that these fire sparsely with precisely timed bursts produced at specific time points in the song motif. It was conjectured that each observed burst represents only a short subset of a continuous chain of activity patterns and, thus, in combination the bursts cover the entire duration of the song (e.g., Fee et al., 2004). This interpretation has been referred to as the “clock hypothesis” of the song system (Troyer, 2013).

Recently, an alternative coding strategy by HVC neurons has been proposed, where the sparse bursts of action potentials in RA-projecting HVC neurons are not viewed as an under-sampled set of recordings of a continuous representation of time. Instead, they are viewed as sparse sets of bursts that code for events in the motor control sequence that are associated with key transitions in the acoustic structure of the behavioral output (Amador et al., 2013). This alternative interpretation has been referred to as the “gesture-transition” hypothesis (Troyer, 2013). This observation poses a challenge to the “clock hypothesis,” which assumes a pre-motor function of HVC in that each burst is assumed to provide instruction to be executed into acoustic behavior after a delay. This delay is required for the signals to pass through the downstream nuclei of the circuit and the execution of movement by muscles. If the bursts in HVC projection neurons occur temporally close to significant motor instances (like the beginning of the syllables), it is tempting to conjecture that there is a relationship between these events. However, if the burst occurs simultaneously with the acoustic gesture, causality seems to be violated. In a top down view of the architecture of the song system, this paradox is difficult to resolve. It has been stressed, however, that the song system is a highly interconnected network with significant bottom up connectivity between the brainstem and song control areas in the telencephalon. Because microstimulation delivered to PAm (a pre-motor area in the lateral medulla that drives inspiration) during singing causes disruptions of song sequencing, these connections have been suggested to play a critical role in the execution of the motor program for singing (Ashmore et al., 2005, 2008). Is it possible to have sparse activity in the telencephalon concurrent with significant motor instances in such architecture?

In order to address this question, we built a model whose variables are the average activity (Hoppensteadt and Izhikevich, 1997) of different neural populations of the song system. The model's architecture is designed to incorporate observations in several areas of the song motor control circuit and we therefore explicitly represented the telencephalic song-control nuclei HVC and RA as well as the thalamic nucleus Uvaeformis (Uva) which connects PAm to HVC. The difficulty for obtaining electrophysiological measurements during singing from respiratory brainstem nuclei, such as RAm which control expiratory drive, prevents us from making more specific associations to these areas and we therefore define it in our model as a generic expiratory related area (ER). In this way, one variable controls expiratory activity during song production, and we require that its activity fits the respiratory patterns observed during song in the canary (Trevisan et al., 2006; Alliende et al., 2010).

**Figure 2:**
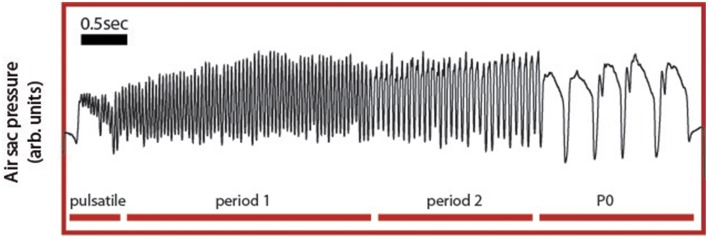
**Experimental respiratory patterns of a canary song**. During song, the air sac pressure is monitored through a cannula inserted through the abdominal wall into the anterior thoracic air sac, which is connected to a miniature piezoresistive pressure transducer. Four basic respiratory patterns form the canary song: the pulsatile, consisting of small pressure fluctuations mounted on a DC value; the P1 (or period 1) patterns, which consist of almost harmonic fluctuations; the P2 (or period 2) patterns last approximately twice as the P1 patterns and typically display a relative minimum during the expiratory gesture. Finally, the P0 patterns are long expiratory gestures characterized by a brief pulse followed by a long expiratory gesture presenting a slow decay until the abrupt end of the pulse.

Song arises from combined activity between the respiratory system and syrinx and we therefore choose ER activity as a key variable in our model because it controls many of the temporal and acoustic features of song. Specifically, the syrinx, which is a bipartite structure between the bronchi and the trachea that holds two pairs of internal labia, modulates the airflow generating sound waves. The configuration of this device can be controlled by the activation of specific muscles, whose contraction is ultimately transduced into acoustic modulations of the generated sounds (Laje and Mindlin, 2005). Because the generation of sound requires establishing airflow between the vibratory tissue, the labia, a bird has to exquisitely coordinate respiratory and syringeal muscles to produce specific acoustic features. Therefore, a central aspect of the motor control of birdsong production is the capacity to generate diverse respiratory rhythms, which determine the coarse temporal pattern of song. The neural mechanisms that underlie this diversity of respiratory gestures and the resulting acoustic syllables are largely unknown. Yet, an interactive model has been proposed, where these motor instructions emerge from the non-linear interaction between timescales of different components of the motor control network (Alonso et al., 2009; Goldin et al., 2013). Support for this integrative view comes from the specific shapes of pressure patterns used to generate the different syllables found in canary song (Figure 2). In canary song, respiratory patterns generating different syllables are highly characteristic and can be reproduced using a simple model (Alonso et al., 2009; Goldin et al., 2013).

The success of the presented model in reproducing the measured respiratory patterns and its specific predictions on the timing of HVC activity during their production, which are consistent with recently reported observations (Amador et al., 2013), support a model for control of vocal production by a circular network rather than by a simple a top-down architecture.

## Materials and methods

Experiments were performed using adult male canaries in accordance with a protocol approved by the University of Buenos Aires, (FCEN-UBA) Institutional Animal Care and Use Committee (C.I.C.U.A.L.).

To record respiratory activity, we monitored subsyringeal air sac pressure through a flexible cannula (silastic tubing, o.d. 1.65 mm), which was inserted through the abdominal wall into the anterior thoracic air sac under isoflurane anesthesia. The free end of the cannula was connected to a miniature piezoresistive pressure transducer (Fujikura model FPM-02PG), which was mounted on the bird's back (for a more detailed description see Goller and Suthers, 1996). The voltage from the transducer was amplified and recorded with a data acquisition device (National Instruments BNC2110). Typically, birds start singing 1 or 2 days after the surgery. Songs and the pressure transducer signal were recorded continuously and simultaneously.

**Figure 3:**
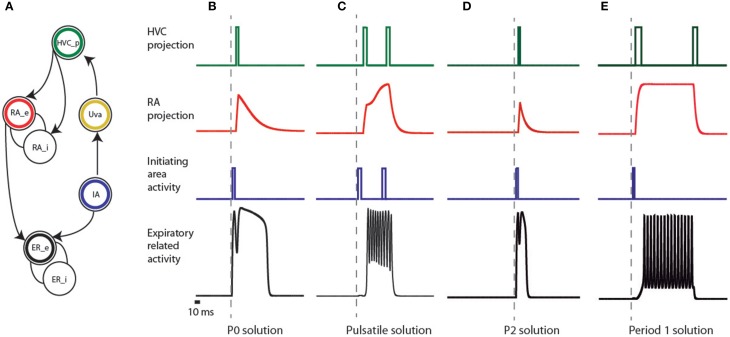
**Elements of the empirical model of the song system and activities of the simulated patterns. (A)** connectivity network of different brain regions of the song system. The expiratory system is modeled as two coupled populations, sketched as interconnected circles at the bottom of the figure, and labeled as ER_e and ER_i. The circle with a black ring represents the excitatory part (ER_e), and the other the inhibitory one (ER_i). In our model, a brainstem area (represented by the circle enclosing a blue ring, labeled IA) is responsible for the initial burst received by both the ER area and nucleus HVC (represented by the circle with the green ring). The passage from the brainstem to the telencephalic structures is represented by the activation of Uva (yellow ring), delayed with respect to the burst in IA. Further processing of this burst is performed in the nucleus RA, which we model by two neural populations: an excitatory (red ring labeled RA_e) and an inhibitory one (labeled RA_i). The input of these populations is the activity of the area representing HVC, and the activity of the excitatory population is sent to ER. **(B–E)** represent the activities produced at the different areas of the model (following the color code of the network) that reproduce the observed expiratory patterns used by canaries in Figure 2, in such a way that HVC is sparsely active, simultaneously with significant motor instances of these patterns. In the framework of additive models, the variables represent the averaged activity of local populations of neurons, i.e., the average number of action potentials generated by the neurons in the population. In this representation, the square pulses consequently denote bursts.

Empirical models for the nervous system arise when we search for a simple dynamical system reflecting one or more important physiological observations (Hoppensteadt and Izhikevich, 1997). In our case, we want to test the hypothesis that it is possible to reproduce the shapes of the air sac pressure patterns in area ER (Figure 3) of the nervous system, while HVC displays sparse activity at significant motor instances.

We built our empirical model in a modular way. The variables are the averaged activities of the neurons in a set of interconnected areas (Hoppensteadt and Izhikevich, 1997). The dynamics for these variables will be ruled by a simple time continuous additive neural network model of the form:

$$\frac{dx_{i}}{dt}\;=\;-x_{i}+S\left(\rho_{i}+\sum_{j}a_{ij}x_{j}\right),$$

$$S(x)\;=\;\frac{1}{1+e^{-x}}$$

where *x_i_* describes the averaged activity of the neurons in the *i^th^* area, ρ_*i*_ the external input to the *i^th^* area, and *a_ij_* describe the connectivity between the regions. The connectivity parameters describe both the strength of the connections as well as the size of the driving populations. The additive model implements the observation that the more excitatory input a neuron receives, the more active the neuron will be.

Previously we reported that a simple model could reproduce the pressure patterns used by canaries during song production (Trevisan et al., 2006; Alonso et al., 2009; Amador and Mindlin, 2014). This model consisted of a neural motif with an excitatory population, an inhibitory population, and a simple time periodic input. Following this work, the first building block of our model is an excitatory population coupled to an inhibitory one, and we require that the activity of the excitatory population fits the measured pressure patterns. In Figure 3A, these two populations are ER_e and ER_i.

One of the significant motor instances mentioned in Amador et al. (2013) is the start of the syllables. One way to account for activity in the telencephalic nucleus HVC that occurs simultaneously with this onset is a *common input* to the ER area and HVC. In our model, this common input is provided by a neural population that we call initiating area (IA), whose activity consists of bursts of spikes that we represent mathematically as a square function a few milliseconds long (see Figure 3A, blue circle labeled IA, and blue traces in Figures 3B–E). We assume that this IA is somewhere in the brainstem. The rationale for this hypothesis is that non-oscine birds lack the complex telencephalic structures found in oscines and yet are capable of generating song-like vocalizations. In oscine birds, brainstem activity is conveyed to the telencephalic structures through the thalamus, similar to efferent copies that are conveyed to cortical or cortical-like areas in other systems (Wolpert et al., 2001). We represent participation of this thalamic pathway by the activation of Uva (Figure 3A yellow circle labeled Uva), delayed with respect to the burst in IA. The green circle represents the nucleus HVC, and we simulate its activity by a burst that is mathematically expressed as a square function 10 *ms* wide, delayed with respect to the burst in the thalamic area. In this way we ensure that the burst in HVC and the start of activity in the expiratory related areas will occur approximately at the same time.

The challenge for this model is whether the further processing of this burst by the nucleus RA and its output that forces the ER area are capable of eliciting features in the respiratory activity that matches those found in recorded air sac pressure during song. In order to test this hypothesis, we model the area representing RA by two neural populations: an excitatory (RA_e in Figure 3A) and an inhibitory one (RA_i in Figure 3A), following the neural architecture reported in the literature (Spiro et al., 1999). The input of these populations is the HVC activity, and the activity of the excitatory population is sent to the ER area as an input ρ.

Mathematically, this model is represented as follows:

(1) $$\begin{array}{l} \frac{de_{er}}{dt}\;=\;149.5(-e_{er}+S(-7.5+\\ \;\alpha_{eer,ra}e_{ra}+\alpha_{eer,F}F+10e_{er}-10i_{er}))\\ \frac{di_{er}}{dt}\;=\;149.5(-i_{er}+S(-11.5+\\ \;\alpha_{ier,ra}e_{ra}+10e_{er}+2i_{er}))\\ \frac{de_{ra}}{dt}\;=\;20(-e_{ra}+S(\rho_{e,ra}+\alpha_{era,Fd}F_{delayed}+\\ \;\alpha_{era,Fd2}F_{delayed2}+\alpha_{era,ra}e_{ra}+\beta_{era,ra}i_{ra}))\\ \frac{di_{ra}}{dt}\;=\;20(-i_{ra}+S(\rho_{i,ra}+\alpha_{ira,Fd}F_{delayed}+\\ \;\alpha_{ira,Fd2}F_{delayed2}+\alpha_{ira,ra}e_{ra}+\beta_{ira,ra}i_{ra})) \end{array}$$

where the variables *e_er_, i_er_, e_ra_, i_ra_* stand for the excitatory and inhibitory populations in the ER area and RA, respectively. *F* is a square function, and stands for the activity in the IA. Notice that it constitutes one of the inputs to the ER area. *F_delayed_* is a square function as well, but one that is delayed with respect to *F*. In our simulations, both *F_delayed_* and *F_delayed2_* stand for bursts of activity in HVC. As the pulses represent burst of activity, the amplitude of the square function was set equal. The height of the pulses was set to 10 (*arb. units*) for all the simulated patterns. These delays were introduced to account for the processing taking place in the thalamus. Otherwise, transmission delays between directly connected nuclei were neglected, which is the usual approximation in additive models (Hoppensteadt and Izhikevich, 1997).

## Results

Canaries generate different song syllables with respiratory patterns that can be classified using singular value decomposition (SVD) into four basic patterns (Trevisan et al., 2006; Alliende et al., 2010). Pulsatile patterns consist of sustained expiratory pressure pulses, which display small modulations where each peak corresponds to a sound pulse. In Figure 2, a pulsatile sequence is followed by a sequence of period 1 pattern (P1), which consists of fluctuations that are almost harmonic in nature. In contrast a period 2 (P2) is a pattern in which pulses are approximately twice as long as those of the P1 and where each expiratory pulse typically displays a pressure modulation with a relative minimum. Finally, period 0 patterns (P0) are sustained expirations that are used to generate tonal whistles. They are long expiratory gestures that typically contain a brief pressure peak followed by a second, long expiratory gesture with a slow decay until the abrupt end of the pulse. The model proposed above displays solutions reproducing these patterns. The time series data used in Figure 2 are included in the Supplementary Materials.

### P0 solutions

P0 solutions can be reproduced in the following way (Figure 3B). The ER area is built out of two populations: an excitatory (black circle labeled ER_e) and an inhibitory one (open circle labeled ER_i). Their activities are *e_er_* and *i_er_*, ruled by the first two equations in Equation (1) (see Materials and Methods). In the framework of additive models, the variables represent the averaged activity of local populations of neurons, i.e., the average number of action potentials generated by the neurons in the population (Hoppensteadt and Izhikevich, 1997). The excitatory population in the ER area first receives a brief pulse of activity of 20 *ms* (represented by *F* in Equation 1) from the IA, represented by the blue circle in Figure 3. The pulse is also sent upstream to the cortical-like areas, arriving at HVC (green circle). This is then conveyed to RA after a 10 *ms* delay through the HVC-RA projection neurons (represented by *F_delayed_* in Equation 1). RA has a set of excitatory and inhibitory populations that process the input from HVC and generate an output, which constitutes a second drive to the ER area of the song system (notice *e_ra_* within the arguments of the sigmoid function, in the first equation of Equation 1). The parameters defining the internal connectivity of RA and its connectivity with other brain regions were chosen so that RA activity (as defined at the beginning of this section), after the input from HVC, consists of a sharp growth followed by an exponential decay (the red time trace in Figure 3B). In this way, the ER area receives two inputs: a direct one, and a processed copy of that input from the telencephalon. The copy might be processed by the contralateral nuclei. In that case, the brief peak of the expiratory pulse and the longer pulse give rise to sounds uttered by the opposite sides of the syrinx. In this way, the model allows a reproduction of the shape of the respiratory gesture, including (1) sparse activity in HVC and (2) a precise prediction on the timing of the activity of the projection population in HVC, i.e., right after the brief pulse in the expiratory gesture.

The parameters of the expiratory area are set to: (α_eer, F_, α_eer, ra_, α_ier, ra_) = (1, 10, 0). The parameters of the RA network are: (ρ_e, ra_, α_era, Fd_, α_era, Fd2_, α_era, ra_, β_era, ra_) = (−3, 5, 0, 6, −3) and (ρ_i, ra_, α_ira, Fd_, α_ira, Fd2_, α_ira, ra_, β_ira, ra_) = (−6, 0.05, 0, 6, 6).

### Pulsatile solutions

Pulsatile pressure patterns consist of small amplitude oscillations superimposed on a sustained expiratory pressure pulse. In Figure 3C we display a plausible mechanism for their generation. The ER area receives a pulse of 50 ms from the IA, to which it is weakly coupled. A copy of this activity is sent to HVC. We assume that this first pulse has an excitatory effect on the excitatory population of RA (RA_e in Figure 3A), and a second peak in HVC is assumed to project to the inhibitory population in RA (RA_i in Figure 3A). The result is a finite amount of time in which RA is active, driving the expiratory related area into an oscillating regime.

In this model, activity of HVC projection neurons is required right before the beginning and right before the end of the pulsatile segment. We are not providing a model for HVC, therefore the second burst (represented by the pulse *F_delayed2_*, and acting as input at the inhibitory population of RA) could either be the result of HVC dynamics, or it could be a delayed response to activity in the IA. In this implementation, both of these bursts originate at the IA, and the time interval between *F_delayed2_* and *F_delayed_* is the duration of the pulsating segment. In this implementation of the model, we are not considering additional periodic activity in HVC, although similar solutions would be obtained provided its frequency is similar to the one of the oscillations induced in the respiratory network.

The parameters used in the simulations are set to: (α_eer, F_, α_eer, ra_, α_ier, ra_) = (0.25, 10, 6). For the RA network the parameters are set to: (ρ_e, ra_, α_era, Fd_, α_era, Fd2_, α_era, ra_, β_era, ra_) = (−5.25, 15, 0, 10, −10) and (ρ_i, ra_, α_ira, Fd_, α_ira, Fd2_, α_ira, ra_, β_ira, ra_) = (−12, 0, 25, 10, 2).

### P2 solutions

This model can also reproduce P2 patterns (Figure 3D), which are expiratory pulses displaying a local minimum with a duration approximately twice that found in the simplest, almost harmonic patterns (P1). The relative amplitude of the local maxima can vary in different syllables generated by P2 solutions. These pressure patterns are used in vocalizations involving sequential use of the two sides of the syrinx, similar to bilateral contributions in syllables generated by P0 solutions (Suthers et al., 2004). As in P0 solutions, we make the assumption in the model that the part of the pulse between its beginning and the local minimum is used to generate sound with one of the two sound sources, while the rest of the pulse is used to generate sound with the second source. In our implementation of the P2 solution, the rapid growth of the peak is the result of the driving from the IA, a pulse of 20 *ms*. After this brief pulse of activity, the activity decays until the input from the telencephalon arrives, 22 *ms* after the first one. The difference between these solutions and the P0 is that the activity from RA is not strong enough to drive the ER area into the “on” fixed point (compare the red traces for B and D in Figure 3), and therefore its contribution to the expiratory pattern is a small modulation in pressure. The implementation shows the plausibility of generating a time trace with the shapes of the measured patterns, together with sparse activity in HVC at significant instances of the motor gesture; in this case, at the beginning of the syllable and the transition between the two sources. As before, the key to reproducing this pattern is the dual forcing that arrives at the ER area, (1) the direct one from the IA and (2) the one processed by the telencephalon.

In this implementation, we used (α_eer, F_, α_eer, ra_, α_ier, ra_) = (1, 10, 0) for the expiratory system. The network describing RA for the generation of this syllable is (ρ_e, ra_, α_era, Fd_, α_era, Fd2_, α_era, ra_, β_era, ra_) = (−7, 2, 0, 3.5, −5) and (ρ_i, ra_, α_ira, Fd_, α_ira, Fd2_, α_ira, ra_, β_ira, ra_) = (−4.5, 0.05, 0, 16, 6). The change in these parameters affects the shapes of the pressure pattern and thus simulates the observed differences in relative amplitude between maxima in the recorded pressure patterns.

### P1 solutions

P1 solutions occur in canary song typically right after syllables arising from pulsatile pressure patterns. For this reason, we looked for a mechanism that represents the smallest departure from the one generating the pulsatile patterns (Figure 3E). Pulsatile solutions were found with activity in RA displaying continuously increasing amplitude (see Figure 3C), while P1 solutions were obtained when RA reached and sustained constant asymptotic amplitude. It is parsimonious to assume that a continuous process would start with pulsatile solutions and with minimal adjustment change to a P1 pattern, in agreement with sequences observed in canary songs (see Figure 2). In this way, a constant input into the expiratory related area allows generating a periodic expiratory activity. This does not exclude the possibility of reinforcing this periodic behavior with a periodic input arriving from HVC.

In the simulations of Figure 3E, the parameters used are (α_eer, F_, α_eer, ra_, α_ier, ra_) = (0.25, 4.65, 4.5) for the expiratory system. For the RA network we used (ρ_e, ra_, α_era, Fd_, α_era, Fd2_, α_era, ra_, β_era, ra_) = (−5.25, 35, 0, 10, −10) and (ρ_i, ra_, α_ira, Fd_, α_ira, Fd2_, α_ira, ra_, β_ira, ra_) = (−12, 0, 25, 10, 2). The pulses are 40, 140, and 100 ms long for *F, F_delayed_* and *F_delayed2_* respectively. C code for the numerical integration of the equations along with the simulated time series described in this section is included in the Supplementary Material.

### Reproducing cooling experiments

Support for a paradigm in which the telencephalon takes over downstream circuitry has been provided by an experiment performed in zebra finches, in which mild cooling of HVC led to song stretching (Long and Fee, 2008). Recently, work in canaries also showed song stretching under mild cooling, and a progressive restructuring of song under further cooling. Particularly, long pressure patterns (like the P0 patterns discussed in this work) disassembled into patterns with shorter pulses. This is an observation that cannot be accounted for by a top down model of the song system. In this section we show that the circular model parsimoniously accounts for the breaking of the long syllables reported in canaries.

In order to introduce the effect of cooling into our simulations, we assumed that locally reducing the temperature in HVC would have at least two effects. First, it would slow down the biophysical processes controlling the dynamics within HVC. This would specifically imply the stretching of the bursts that could be elicited in the neurons of this nucleus. Second, it would slow down recurrent inputs from Uva to HVC. In this way, the simulations of patterns for normal and lowered HVC temperatures would involve the same circuit parameters, they would be started by identical pulses from the initiating area, but in the second case, the bursts in HVC would be both longer (first effect) and would start slightly later than in the normal temperature case (second effect). The later effect will be particularly important in those patterns whose shapes preserve the fingerprints from both the direct brainstem drive as well as from the drive imposed by the telencephalon. For this reason, we will discuss the P0 patterns in detail.

**Figure 4:**
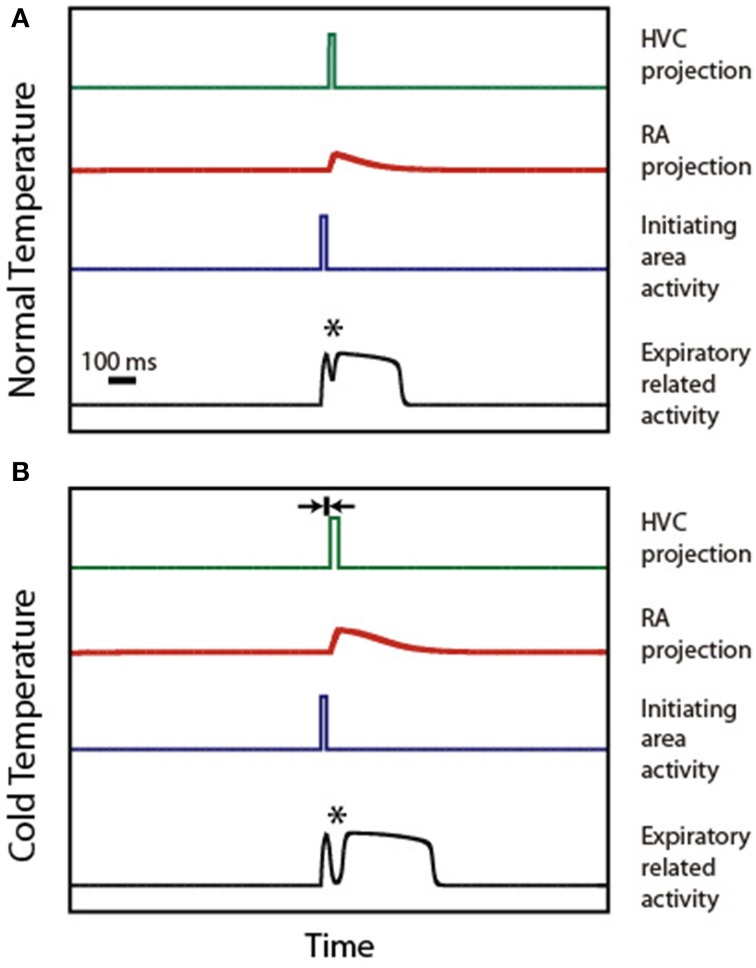
**Syllable stretching and breaking under HVC cooling. (A)** displays the time traces of activity in HVC, IA, RA, and ER during the production of a P0 solutions under normal conditions. **(B)** displays the time traces of the activities in the same brain regions, assuming that the burst representing HVC activity starts slightly delayed and stretched. The rationale for the delay is the slowing down of the synapses involved in the connection between Uva and HVC. The rationale for the stretching is the slowing down of the internal dynamics within the cooled HVC. When both effects are present, the model reproduces the observed stretching and breaking of P0 patterns. The asterisk indicates the position of the minimum.

Figure 4 illustrates the mechanism of syllable deformation according to the circular model. The first panel displays a simulation for the normal temperature case. The parameters are set so that the expiratory related activity is a P0 pattern, i.e., it shows a small peak, a local minimum, and a final longer pulse. The second panel displays the simulations for the cooled HVC case. The second time trace in this panel (representing the activity of the initiating area) is identical to the one used in the upper panel. Yet, we use for HVC activity a longer pulse than in the normal temperature case (15 ms longer), starting slightly later (5 ms). This additional delay is represented in the second panel as the width of the small segment bounded by arrows. The other parameters are (α_eer, F_, α_eer, ra_, α_ier, ra_) = (1, 10, 0), (ρ_e, ra_, α_era, Fd_, α_era, Fd2_, α_era, ra_, β_era, ra_) = (−3, 5, 0, 6, −3) and (ρ_i, ra_, α_ira, Fd_, α_ira, Fd2_, α_ira, ra_, β_ira, ra_) = (−6, 0.05, 0, 6, 6).

In both simulations, an asterisk indicates the local minimum. This feature of the pattern emerges from adding the (decaying) activity of the respiratory gesture induced by the direct action of the initiating area, and the (growing) activity arriving from the telencephalon. The decrease of the value of the pressure at the minimum leads to the “breaking” of the pattern as the minimum reaches phonation threshold: the initial pattern becomes a set of two separate pressure pulses. This decrease originates in the temperature-induced increase in processing time in HVC. In fact, simulations involving only the first effect (burst stretching without onset delay) lead to stretching without breaking of the pressure patterns. It is the second effect (onset delay) that generates the separation between the directly driven expiratory pulse and the second part of the pulse which is driven by the telencephalon. In our simulation, the longer pulse in HVC (first effect) also leads to a longer period of activity in RA, resulting in a pattern that was also stretched.

## Discussion

Here we present an operational model that proposes a neural mechanism for how the apparently paradoxical observation of concurrent bursting activity of HVC projection neurons and changes in behavioral state (Amador et al., 2013) could represent a plausible motor strategy in the song control circuitry of songbirds. We propose song production in the canary as a suitable model, because its song is composed of relatively simple and repeated syllables that present relatively long and regular intervals between acoustic state changes such as syllable onset and offset and switching between left and right sound sources. These features make canary song more amenable to modeling than the temporally more complex and less regular features of zebra finch song. The model does not only account for the observations of HVC neurons and the respiratory patterns of song, but it also makes clear predictions for information flow in the song circuitry that can be tested.

In this model, the activity of the expiratory related area receives two inputs. The first input originates from a neural population (here called IA), and the second represents a copy of this initial drive processed by telencephalic centers and therefore contains a time delay. The model assumes integrated processing in different areas in contrast to an alternative view of the song system in which HVC output dictates all the time scales in the behavioral output (Fee et al., 2004; Long and Fee, 2008; Andalman et al., 2011). The integrated processing that is distributed across all nuclei of the circuitry in this model emphasizes that telencephalic input modulates structures that are involved in generation of all vocal behavior, including non-learned vocalizations for which telencephalic areas are not required. Such a layered organization in song motor control is important in light of the evolutionary progression from vocal control based entirely on mid- and hindbrain structures in non-vocal learners to one where telencephalic circuitry is involved in producing part of the repertoire and in its acquisition during song ontogeny. Considering this evolutionary trajectory is even more important in light of the vital functions (respiration) of the preexisting, evolutionary older neural circuitry into which vocal control gets integrated. It is highly unlikely that telencephalic input evolved to “usurp” this circuitry.

Correspondence of specific nuclei of the song system with the various areas in the model cannot be established in all cases at the present time, although we suggest areas based on their currently known roles (HVC, Uva, and RA are explicitly considered). The sparse activity in nucleus HVC, synchronized with significant motor events, is assumed as a hypothesis in this work. The representation of the song system as a circular, highly interconnected set of brain areas was advanced by Schmidt and collaborators (Ashmore et al., 2005) and others (Gibb et al., 2009). The mapping of nuclei of the song system onto the architecture used in our model is more firmly established by known cytoarchitecture and neural behavior for some areas than others. Precise mapping for nuclei in the brainstem is more difficult because recordings in those nuclei are only available for sleep or anesthetized conditions but not in spontaneously singing birds. However, we make suggestions based on our current understanding of the respective roles. The excitatory and inhibitory populations for respiratory control in our model can most likely be mapped onto the analogous populations in RAm, since this nucleus is associated with expiratory related activity. Whether the IA in our model is a subpopulation of either PAm or DM is not clear. In any case, the model predicts that at least one of these is likely to present neurons branching to thalamus and medulla. Both connect to telencephalic areas through the thalamic nucleus Uva. This connection starts the indirect path from the IA via the telencephalic areas back to the ER area.

In the present model, HVC receives inputs initiated at the brainstem. However, despite its proposed role as only playing one part in the integrated and distributed motor control of song production, HVC activity is necessary and critical for reproducing specific features in the respiratory patterns. This role is therefore still consistent with data from experiments in which HVC has been lesioned and song patterns are disrupted (Halle et al., 2003). If the contribution from RA to the ER area were interrupted, many of the pressure gestures described in this work would be seriously affected. For example, the large pulse in the P0 solutions would not occur. The brief, direct input from the IA to the ER area would only lead to a brief pulse. It is interesting to conjecture that the relative simpler vocal patterns found in non-oscines correspond to less complex gestures that are generated only with the brainstem without the additional input from the copy processed in the telencephalon. The learned aspects of song production therefore would be represented in this part of the input to the expiratory related activity.

In the present model we make no attempt at modeling the dynamics in HVC after arrival of the input from the thalamus (Daou et al., 2013; Basista et al., 2014). It is likely that the subsequent activity, needed for some syllables to stop the expiratory patterns (Figures 3C,E), emerges from the interaction between excitatory and inhibitory populations of neurons. It is also plausible that P1 and pulsatile solutions involve a periodic activity in HVC that follows the initiating pulse. It is noteworthy that once the sequence of activity patterns in HVC is learned, it could be simply generated without the input from IA. In that case, the patterns would be slightly different, with the most significant differences occurring during the first few milliseconds of the gesture.

Cooling of specific brain areas has been used to investigate the temporal coding of birdsong production (Long and Fee, 2008). The rationale behind these experiments is that a nucleus cooled by a few degrees Celsius would exhibit slower neural dynamics. We mentioned that period 1 and pulsatile patterns could be generated with or without additional periodic activity in HVC, and further experimental work is required to decipher the actual mechanism. In the first case, assuming that cooling lowers the frequency of the periodic activity in HVC, the locking of the respiratory oscillations leads to stretching of the pressure patterns (Goldin et al., 2013). Without additional forcing, it is still the case that lower HVC activity leads to lower RA activity, which gives rise to oscillations of lower frequency in the ER area (Amador and Mindlin, 2014). Both mechanisms are compatible with either a top down description of the song system or the circular model presented in this work. It is in the description of the deformations of P0 patterns under cooling that the circular model departs from predictions in a top down paradigm. By increasing the time between the brainstem forcing and telencephalic forcing to the ER area, the circular model recovers the breaking of long respiratory patterns under cooling. This is an observation that a top down paradigm cannot account for. In this way, the circular model is not only compatible with the breaking of syllables but it importantly also predicts that patterns that break do so at specific locations. These locations are consistent with patterns seen in the experimental data (Goldin and Mindlin, 2013).

The advantage of an operational model, implemented through differential equations, is that it allows performing specific predictions on the timing of the signals in the different areas. These are needed in order to reproduce the shapes of the recorded respiratory time traces (air sac pressure signals). Beyond these predictions, the model already accounts for unilateral sound production in pulsatile and P1 solutions, and sequential contributions from the two independently controlled sound sources in P0 and P2 solutions. These solutions do require both the direct input from ipsilateral brainstem drive and the processed copy through the telencephalon, which requires a contralateral connecting signal, possibly via Uva (Halle et al., 2003). This second signal arrives at the respiratory areas, which are always synchronized. In this way, some syllables require precise coordination between the activities in both brain hemispheres. In our model, the existence of an IA responsible for both activities provides a parsimonious coordination mechanism.

In recent years, the song system has been interpreted as a top down circuitry where the telencephalon controls all the downstream structures. Here we have shown that a circular architecture can reconcile the observed respiratory shapes used by canaries during song production with the reported observation that activity of projection neurons in HVC occurs simultaneously with significant motor instances such as syllable onset and offset. A circular architecture provides an explanation for this observation by supposing a common source for both activities. The expiratory control area therefore receives both signals from the brainstem as well as from the telencephalon. This model of complex motor control presents the first example where the specific temporal characteristics of a motor pattern result from a processed efferent copy as a secondary input. It is highly likely that complex motor control of different behaviors may use conserved neural mechanisms, and this model may therefore provide a useful theoretical framework for interpreting and testing motor control strategies in other complex motor systems.

### Conflict of interest statement

The authors declare that the research was conducted in the absence of any commercial or financial relationships that could be construed as a potential conflict of interest.
