# Supplementary material for: A circular model for song motor control in Serinus canaria
Source: Front Comput Neurosci. 2015 Apr 7;9:41. doi: 10.3389/fncom.2015.00041 (PMC4387923; doi:10.3389/fncom.2015.00041)
Supplement: Supplementary file 1 [file DataSheet1.ZIP › Circular_supplementary/readme.pdf]

Supporting information

pressure.dat contains the time series data (in ascii) of the pressure patterns shown in Figure 2. Columns represent time (sec.) and pressure (arb. units).

pulsatile.c, *p1.c*, *p2.c* and *p0.c* are the codes in C for the numerical integration of the equations of the model. Compilation is performed by the following command:

```
gcc x.c –lm –o out; ./out
```

where x.c represents any of the provided c files.
The columns of the output files correspond to the time evolution of the different variables of Equations 2. The first two columns are time and expiratory-related activity, as shown in Figure 3 for the different patterns: *pulsatile.dat*, *p1.dat*, *p2.dat* and *p0.dat*.
